# Supplementary material for: The Tat Protein of HIV-1 Prevents the Loss of HSV-Specific Memory Adaptive Responses and Favors the Control of Viral Reactivation
Source: Vaccines (Basel). 2020 Jun 4;8(2):274. doi: 10.3390/vaccines8020274 (PMC7349931; doi:10.3390/vaccines8020274)
Supplement: Supplementary file 1 [file vaccines-08-00274-s001.pdf]

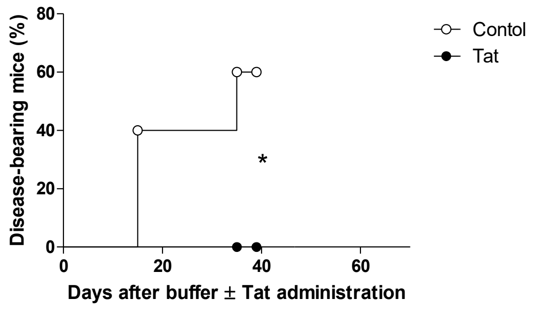

Figure S1. Tat exerts therapeutic effects on previously acquired HSV-1 infection. Mice were infected by the intravaginal route with 10^3 PFU/mouse of HSV-1 and treated with Tat (or buffer) 44 days later. Mice were observed twice per week after treatment to monitor the appearance of signs of disease. The Kaplan-Meier test was used to estimate the probability of clinical manifestation (n = 5 per group). * $p <$ 0.05.

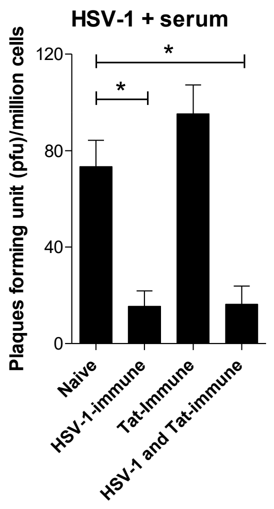

Figure S2. Anti-Tat antibodies do not interfere with HSV-1 replication. HSV-1 (10^{-4} PFU) was pre-incubated with 2 µl of sera from naïve, HSV-1-immune, Tat-Immune or HSV-1- and Tat-immune mice for 1 hour at 37°C under mild agitation. Samples were then added to monolayers of Vero cells (400000 cells/well) at 10^{-6} PFU in 12-well plates. After incubation for 1 hour at 37°C, the medium was replaced with 1 ml of methylcellulose. After further 23 hours of incubation at 37°C, cells were fixed and stained and plaques counted. Bars represent mean ± SEM of 5-7 experiments. Results from HSV-1- and/or Tat-immune sera were compared with HSV-1 pre-incubated with a naïve serum using the Wilcoxon signed rank test. * $p < 0.05$.

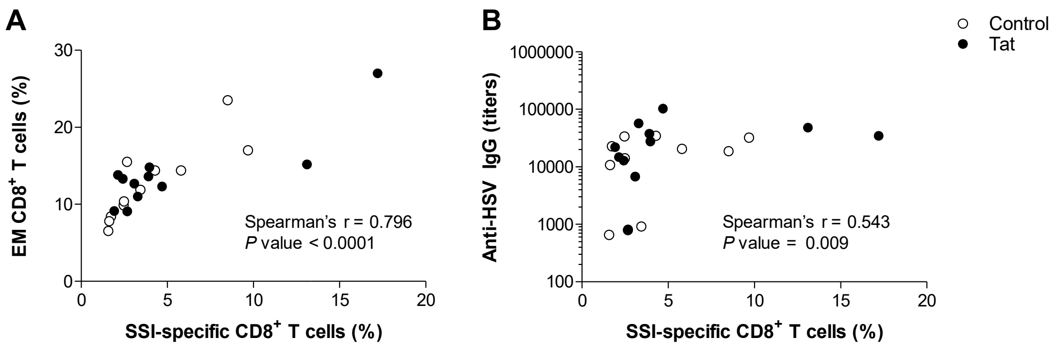

Figure S3. Correlation between HSV-1-specific CD8^{+} T cells, effector memory (EM) CD8^{+} T cells and HSV-1-specific humoral immunity. The percentage (%) of circulating SSI-specific CD8^{+} T cells measured at days 60 and 108 p.i. (days 16 and 64 after buffer ± Tat inoculation, respectively) was correlated with the percentages of circulating EM CD8^{+} T cells (**A**) or with HSV-1-specific IgG titers (**B**). Spearman's rank correlation determined statistical significance (n = 11 per group). In the graphs, Spearman's r and p value of combined Tat and Control mice are reported. Values for Tat and Control separated are as follows: r = 0.93, $p < 0.001$ for Control and r = 0.59, $p = 0.06$ for Tat (A); r = 0.41, $p = 0.21$ for Control and r = 0.63, $p = 0.04$ for Tat (B).
